# State policies that promote, and that inhibit, improved public health: An exploratory analysis of paid sick leave

**DOI:** 10.3389/fpubh.2022.1003117

**Published:** 2022-11-17

**Authors:** Douglas A. Wolf

**Affiliations:** Aging Studies Institute, Syracuse University, Syracuse, NY, United States

**Keywords:** paid sick leave, preemption, right-to-work laws, public health, labor unions

## Abstract

The United States has no national requirement that employers provide paid sick leave (PSL) to their employees, despite the many established public health benefits of PSL access. Many states, and some localities, have passed laws requiring PSL within their jurisdictions. Past studies have shown that these PSL mandates are effective in promoting increased PSL access. However, past studies have not considered two other commonly-used state policy initiatives—PSL preemption and right-to-work laws—that could hypothetically influence employers' decisions to provide PSL. During the past few decades, all possible combinations of these policy interventions can be found in one or more U.S. states. This study estimates the combined associations of these 3 policies with PSL access. The estimates support recent research on the positive effects of PSL mandates, but also suggest that PSL preemption and right-to-work laws may have offsetting effects. Failure to take account of these additional policies may lead to an over-estimate of the effectiveness of PSL mandates.

## Introduction

Access to employer-provided paid sick leave (PSL) has been shown to be beneficial to employees and employers, and to improve public health. Workers with PSL are less likely to show up for work when ill (1, 2), are less likely to delay seeking medical care (2), and increase their use of outpatient care (3) while reducing their usage of emergency department care (4). Workers with PSL access have also been found to have higher levels of retirement savings (5). Employers that offer PSL experience reduced rates of occupational injuries and illness (6) and of overall leave-taking (7) among their workforces. Some studies have identified specific forms of illness—influenza (8) and food-borne illness (9), for example—whose prevalence is diminished by employer-provided PSL. Employers also experience benefits in the form of lower rates of both employee separation (10) and a more general indicator—employee turnover—that encompasses hires as well as separations (11). Finally, PSL has been shown to reduce both all-cause mortality and mortality from specific causes among working-age adults (12, 13).

Along with these benefits, providing PSL also imposes costs on employers, who must pay workers for the accrued sick time that they take; also, the aggregate amount of employee time lost to illness may be greater when PSL is provided than when it is not. In March 2020 it was estimated that the average cost of PSL to employers was $0.45 per employee hour worked (14). These costs, in turn, are equal to 2.5–3.3% of employees' wage compensation (15).

Until recently, private employers have not faced any legal requirements to offer PSL benefits. Private-sector fringe benefits, of which PSL is one component, were rare prior to World War II (16), but have grown rapidly since then. In the absence of legal requirements, employers' provision of benefits has been ascribed to bargaining by labor union, a firm's stability and profitability, religious and ethical concerns of corporate leaders, and institutional factors such as the professionalization of human resources personnel (17). The U.S. Labor Department reported on the prevalence of employee benefits for the first time in 1979, at which time 56% of full-time employees in private-sector industries had access to PSL (18). By 2020 this figure had grown to 78% (19).

However, there is no Federal law requiring PSL coverage in the private sector, and as a consequence there remains great variation in PSL coverage by occupation, region, industry, and wage level, among other factors. As a way of broadening PSL access and reducing inequality in access to it, a growing number of U.S. states have begun to pass laws that mandate a minimum (or “floor”) level of PSL coverage (20). The first such PSL mandate was adopted in Washington DC in 2008, and by 2021, 14 states had such laws in place. Several local-level governments—counties or cities—have also passed laws mandating PSL coverage for workers within their jurisdictions (21).

Two recent studies have investigated the effectiveness of PSL mandates with respect to increased employee access. Maclean et al. (15) used restricted-access individual job-level data from the US Labor Department's annual Employee Compensation Survey (ECS) for 2009 through 2017 to estimate the impact of state PSL mandates on PSL access. Using a difference-in-differences (DD) methodology, with additional controls for a worker's union membership and full-time status, they find that on average, access to PSL for workers in PSL mandate states is 12.8% higher than for their counterparts in non-mandate states, a difference that is statistically significant. A second paper, by Callison and Pesko (22), also used restricted-access individual-level data, taken from the National Health Interview Survey for 2005–2018, and considered both state- and local-level PSL mandates. Their DD estimates also find statistically significant increases in PSL coverage attributable to the PSL mandates, ranging from about 8 to over 20 percentage points, in various model specifications.

Both of these recent studies provide strong evidence that PSL mandates produce an increase in PSL coverage. However, neither study takes into account other state-level policies that might influence PSL access, and that might even counteract the positive effects of the mandates on access. Variation within both the treatment-group and the comparison-group jurisdictions along relevant policy dimensions could undermine the validity of the estimated PSL mandate effects. This study considers two such policy domains: PSL preemption and so-called “right-to-work” (RTW) laws.

State PSL preemption laws, which restrict the ability of lower-level governments to impose PSL requirements, are one manifestation of a larger and growing phenomenon whereby states restrict their constituent governments' actions in areas such as environmental, health, and public-safety domains (23). During the period 2009 through 2021, the number of states with PSL preemption laws grew from 1 to 24 (20). PSL mandates can be characterized as a type of preemption, because they establish a floor level of benefits below which local governments cannot depart. Alternatively, states can pass “ceiling” preemption laws that prevent local governments from requiring even higher levels of PSL. The ceiling is often set at zero, effectively ruling out a government-mandated PSL requirement statewide. A few states have passed a PSL mandate (a floor) while simultaneously imposing ceiling preemption, thereby establishing a floor level of PSL benefit within the state but also preventing lower-level governments from going above that level. PSL mandates and ceiling preemption can be viewed either as two separate policy domains, or can be interacted so as to identify three policy regimes (mandate without ceiling, ceiling without mandate, or mandate with ceiling).

Paid sick leave preemption surely inhibits growth in the prevalence of PSL access, but it also may actually reduce the prevalence of PSL access if it induces employers that might otherwise add to their fringe benefit package not to do so. PSL preemption might also contribute to a decline in PSL access, if employment growth is greater in “business-friendly” states—states that have passed preemption laws—than in states with more stringent regulatory requirements. Differential employment growth could result from existing businesses' decisions to relocate, or from the location decisions of new enterprises.

Right-to-work laws, which prohibit workplace contracts that require employees who are not union members to contribute to the costs of union representation, are aimed at reducing union strength and are associated with reduced overall wage levels and with lower levels of employee benefits, including access to paid sick leave (24, 25). Therefore, right-to-work laws could contribute to diminished growth in, or to actual reductions in, the prevalence of PSL access.

The possible effects of PSL preemption and of RTW laws on PSL access, whether individually or in combination with PSL mandates, does not appear to have been studied. This paper presents an exploratory analysis of the three policies, viewing each of the three state-level policies as “treatments.” It uses a straightforward empirical approach and readily available public data sources.

## Methods

### Data

Data from several online sources were combined for this analysis. The outcome variable, PSL coverage, is taken from annual tables published by the US Department of Labor (26). These tables show, for 2009–2021, the percentage of civilian workers with access to PSL in each of 9 Census Divisions. The Divisions contain from 3 to 9 states (27). Thus, there are 9 (divisions) times 13 (years) = 117 observations in the data file.

Online sources provided, for each state, the implementation year for PSL (floor) mandate laws (21), PSL (ceiling) preemption laws (28), and RTW laws (29). For each policy variable (mandate, ceiling, and RTW, respectively) a series of annual indicators was created, where a value of 1 indicates that the policy was present that year, and a zero indicates that the policy was not present. These state-by-year policy variables were aggregated to the Census Division level, using as weights the size of each state's civilian employed population in the relevant year. Population counts came from the Bureau of Labor Statistics' Local Area Unemployment series, which includes counts of employed people (30). The entire data set is included in the Supplementary material.

### Analysis

The data used in this study do not support the widely used DD approach to inferring causality with non-experimental data. Each observation used here represents a collection of states for which, in any given year, some may have, while others have not, implemented one or more of the policies studied. For each division-year observation the three policy variables fall into the 0, 1 interval (inclusive). Thus, there is not an evident “pre-treatment” nor an evident “post-treatment” period for any of the Census divisions. This problem is further complicated by the fact that three distinct treatments are considered, and that in any given year, several combinations of the treatments may have been adopted.

As a consequence, a simple weighted least-squares regression approach is used here. The outcome, PSL coverage, is regressed on three measures of district-level presence of each of the three policies, that is PPSLMAN (proportion covered by a mandate), PPSLPRE (proportion covered by ceiling preemption), and PRTW (proportion covered by a RTW law). In alternate specifications, all possible combinations (i.e., interactions) of the mandate and ceiling variables are entered, as is an interaction between ceiling and RTW In all cases, the treatments were coded to begin in the calendar year after the respective law was implemented, to allow for lags in employers' responses to the policy changes. All regressions also include fixed effects for Census divisions as well as calendar year dummy variables. It should be noted that in appearance, this regression looks just like the two-way fixed effects regression widely used in evaluation research; it is the data, and not the estimating equation, that departs from the usual DD setup. Division-year observations are weighted by the size of the civilian employed population in the division that year. Inference is based on confidence sets and *p*-values obtained using the wild cluster bootstrap algorithm (31). With the highly aggregated data used here, most of the within-division (i.e., between-state) variability in both outcomes and regressors is lost, and there is a precipitous loss of degrees of freedom. As a consequence, the potential for obtaining statistically significant results is greatly diminished. Therefore, the analysis must be viewed as exploratory, and the results as suggestive rather than as definitive.

## Results

**Figure 1:**
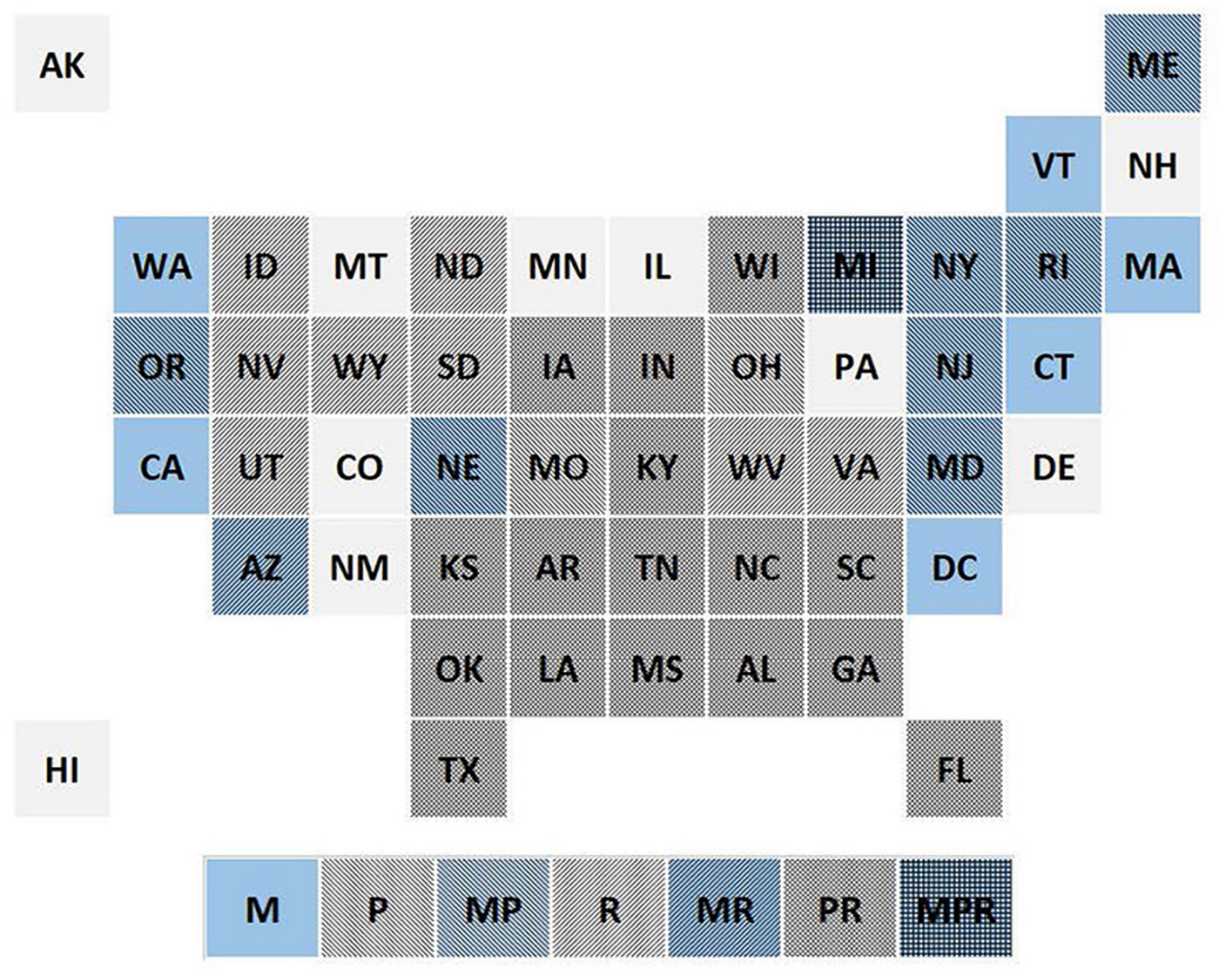
Tile grid map showing the pattern of PSL mandate, PSL preemption, and right-to-work laws, in 2021 (M, mandate; P, preemption; R, right-to-work).

Figure 1 illustrates the spatial pattern of the three policy domains for 2021, in the form of a tile grid map. In this map policies are coded according to their year of implementation. Prior to 2009, just one state (Washington DC) had adopted a PSL mandate, but 21 states had already adopted a RTW law, mostly during the 1940s and 1950s. Additional PSL mandates began to appear in 2012, while PSL preemption first appeared in 2012, with both types of policies spreading thereafter. Five more states also adopted RTW laws beginning in 2012. By 2021, a eight possible combinations of the three binary policy indicators occurred at least once.

Several sets of regression results are reported in Table 1. The first four regressions consider only one of the three state-level policies, in turn; the remaining models address PSL mandates in combination with the other two policies. Model (1) includes only the mandate treatment, and uses only years 2009–2017, the same years used in Maclean et al. (15). The point estimate of the PSL mandate effect suggests an increase in PSL coverage of 8.7 percentage points, a result reasonably close to that obtained in Maclean et al. (15), and well within the 95% confidence interval of the earlier paper's estimate. However, the confidence set for this estimate includes zero. Model (2) uses all available years −2009-2021—and finds a much larger, and statistically significant, mandate effect, a nearly 16 percentage point increase in PSL coverage. The large increase in mandate effects is most likely due to the fact that when adding the more recent years, several additional states have now implemented PSL mandates, and past research has shown that the effect of the mandate on coverage is largest in the first few years of its existence.

**Table 1:** Estimated effects of policy variables on paid sick leave coverage, various specifications.

**Variable**	**(1)^a^**	**(2)**	**(3)**	**(4)**	**(5)**	**(6)**
Mandate	0.087 [−0.078, 0.389]	0.155^*^ [0.019, 0.331]				
Ceiling			−0.114 [−0.246, 0.026]			
Right to work (RTW)				−0.064 [−0.881, 0.796]		0.013 [−0.163, 0.205]
Mandate, no ceiling					0.140 [−0.101, 0.183]	0.141 [−0.105, 0.184]
Mandate with ceiling					−0.026 [−0.169, 0.282]	−0.004 [−0.141, 0.305]
Ceiling, no mandate					−0.042 [−0.076, 0.001]	0.025 [−0.075, 0.208]
Ceiling X RTW						−0.083 [−0.301, 0.023]

Bootstrapped confidence sets in square brackets.

* Bootstrapped p-value < 0.05.

a Equation (1) uses data for 2009–2017; all other equations use data for 2009–2021.

Models (3) and (4) investigate the effects on PSL coverage of ceiling preemption and of RTW laws, respectively, in each case without controlling for the other two policies. In both cases the regression coefficients have negative signs, suggesting that both ceiling preemption and RTW laws reduce workers' access to PSL. Both coefficients are, however, imprecisely estimated.

However, it is clear from Figure 1 that the three policies are not independent of each other, indicating that they should be considered jointly. Model (5) distinguishes the three possible combinations of mandate (or floor preemption) and ceiling preemption. It appears that only when a mandate is not accompanied by ceiling preemption does it increase PSL coverage. Ceiling preemption in the absence of a mandate—that is, a statewide prohibition on governmental requirements that employers provide PSL—appears to reduce PSL coverage (in this case, with a borderline-significant *p*-value of 0.085).

Equation (6) contains interaction effects involving all three policy domains. This regression suggests that a mandate not accompanied by a ceiling has the largest positive effect on PSL coverage, while the combination of ceiling preemption and a RTW law has the largest negative effect on PSL coverage. The other variables included in (6) produce estimates very close to zero, although it must be acknowledged that zero is included in the confidence sets of all the estimated coefficients.

## Discussion

The results reported here support those from two recent papers (15, 22) that found statistically significant increases in workers' access to PSL as a result of state-level PSL mandates. Using aggregated data from the Labor Department's Employee Compensation Survey (ECS) for 2009–2017, the mandate effect is close to the estimate reported in Maclean et al. (15), which used the same survey, in its original disaggregated form, for the same years. The comparability of my estimated PSL mandate to those reported in Callison and Pesko (22) cannot be determined due to non-overlapping years studied.

Using the full 13 years of data and a specification analogous to that in Maclean et al. (15), the effect of a PSL mandate on PSL coverage is positive and statistically significant. However, due to data limitations I am unable to use the specifications, and perform the sensitivity tests, and carry out other robustness checks that have become best practice for supporting claims of causality with non-experimental data. Yet the fact that my results are so close to those previously reported, despite these limitations, is reassuring. This analysis also displays the potential for using easily accessed online data to explore issues that have, to date, required expensive and burdensome procedures to make use of restricted-access data (to use the ECS data, a researcher must first be approved by the Labor Department and, upon approval, travel to one of a limited set of data enclaves to carry out the analysis).

The main point of this analysis, however, is that state adoption of PSL mandates has occurred along with the adoption of other policies—preemption of lower-level governments' ability to mandate PSL provision, and the adoption of right-to-laws—that are expected to have their own, possibly countervailing, consequences for PSL access. If PSL ceiling preemption and right-to-work laws are each considered as an individual “treatment,” each appears to reduce workers' PSL access. In the models that examine each policy in isolation [i.e., (2), (3), and (4)] a PSL mandate appears to have a larger positive effect on access than either of other policies' negative effects. This is to be expected, since the mandates are targeted directly at expanding PSL access, whereas PSL preemption and right-to-work laws have only indirect consequences for the spread of PSL coverage. When all three policies are considered jointly [in equation (6)], a mandate with no ceiling, and a ceiling in combination with a RTW law, appear to have the largest consequences for PFL coverage. However, as already noted, data limitations dictate that these results be viewed as exploratory, and, at best, suggestive. They can, however, serve as a possible roadmap for follow-up research based on individual- or state-level data.

The present analysis also reminds us of a familiar shortcoming of observational studies, namely their need to deal with omitted-variables biases. In this case, a study that addresses whether states' PSL mandates produce increases in workers' access to PSL, but fails to account for either PSL preemption or RTW laws, appears to be subject to omitted variables biases in its estimate of policy impacts. The present study, of course, is not exempt from this problem, inasmuch as there could be additional factors that vary across states, that are correlated with any or all of the three policy variables used here, and that have their own effects on PSL coverage.

In view of the substantial body of evidence supporting the public health benefits of PSL, and the additional evidence that state PSL mandates lead to higher rates of PSL access, activists will presumably want to direct their efforts toward the further spread of these state-level mandates. It is concerning, however, that PSL preemption, which appears to hinder the growth of, and even reduce the prevalence of, PSL access, has been adopted in more states than PSL mandates have been. Faced with this situation, greater attention might be paid to encouraging adoption of a national PSL requirement. It is noteworthy that the U.S. is the only country, among 22 wealthy nations, that lacks a national PSL law (32). This, in turn, is likely to be just one of several factors explaining the fact that the U.S. continues to lag behind many other countries in various public health indicators, including mortality (33).

## Data availability statement

The original contributions presented in the study are included in the Supplementary material, further inquiries can be directed to the corresponding author/s.

## Author contributions

DW was responsible for all aspects of the research.

## Conflict of interest

The author declares that the research was conducted in the absence of any commercial or financial relationships that could be construed as a potential conflict of interest.

## Publisher's note

All claims expressed in this article are solely those of the authors and do not necessarily represent those of their affiliated organizations, or those of the publisher, the editors and the reviewers. Any product that may be evaluated in this article, or claim that may be made by its manufacturer, is not guaranteed or endorsed by the publisher.
